# Machine Learning for Intelligent-Reflecting-Surface-Based Wireless Communication towards 6G: A Review

**DOI:** 10.3390/s22145405

**Published:** 2022-07-20

**Authors:** Mohammad Abrar Shakil Sejan, Md Habibur Rahman, Beom-Sik Shin, Ji-Hye Oh, Young-Hwan You, Hyoung-Kyu Song

**Affiliations:** 1 Department of Information and Communication Engineering, Sejong University, Seoul 05006, Korea; sejan@sejong.ac.kr (M.A.S.S.); habibur@sju.ac.kr (M.H.R.); hidex9852@yahoo.com (B.-S.S.); wlgp5329@naver.com (J.-H.O.); 2 Department of Convergence Engineering for Intelligent Drone, Sejong University, Seoul 05006, Korea; yhyou@sejong.ac.kr; 3 Department of Computer Engineering, Sejong University, Seoul 05006, Korea

**Keywords:** intelligent reflecting surfaces (IRSs), machine learning, multiple input multiple output, wireless networks

## Abstract

An intelligent reflecting surface (IRS) is a programmable device that can be used to control electromagnetic waves propagation by changing the electric and magnetic properties of its surface. Therefore, IRS is considered a smart technology for the sixth generation (6G) of communication networks. In addition, machine learning (ML) techniques are now widely adopted in wireless communication as the computation power of devices has increased. As it is an emerging topic, we provide a comprehensive overview of the state-of-the-art on ML, especially on deep learning (DL)-based IRS-enhanced communication. We focus on their operating principles, channel estimation (CE), and the applications of machine learning to IRS-enhanced wireless networks. In addition, we systematically survey existing designs for IRS-enhanced wireless networks. Furthermore, we identify major issues and research opportunities associated with the integration of IRS and other emerging technologies for applications to next-generation wireless communication.

## 1. Introduction

Wireless communication systems are changing dramatically as a high data rate and quality of service are in soaring demand. Fifth generation (5G) wireless communication has already been deployed and is currently in service in many countries [1]. Therefore, sixth generation (6G) wireless communication has received full attention from the researchers. The 6G wireless networks include an ultra-high data rate, high reliability, global coverage, low latency, high energy efficiency, and high reliability [2]. To meet these requirements, we need more advanced network devices and new techniques for efficient wireless communication. The literature published in recent years suggests that, for 6G, the main ideas are terahertz communication, artificial intelligent and intelligent reflecting surfaces (IRSs), or reconfigurable intelligent surfaces [3,4].

IRS is considered a new paradigm for wireless communication in the 6G [5]. IRS is a thin metasurface made of a dielectric material capable of reflecting electromagnetic (EM) waves in an organized way. Thus, IRS is considered to be a software-controlled smart radio environment that can mitigate multipath problems and is effective for millimeter wave or terahertz communication [6]. In recent years, the application of IRS devices has dramatically increased, owing to its excellent convenience in wireless communication, such as mobile edge computing, simultaneous wireless information and power transfer, enhanced physical layer security, device-to-device communication, mmWave massive multiple-input multiple-output (MIMO), unmanned aerial vehicles communication for smart cities, and intelligent internet of things (IoT) applications for wireless sensor networks [7]. In the state-of-the-art, many studies have been conducted on the design of IRS-aided communication for the different performance matrices [8,9,10]. The study conducted in IRS can be categorized as cascade channel estimation, phase shift optimization, beamforming optimization, multiuser communication, and reflecting metasurface grouping [11,12,13,14].

Machine learning (ML) methods are considered as a revolutionary technology of the branch of artificial intelligence (AI) [15]. ML methods can learn from data and make predictions for future events. Recently, ML-based approaches have been widely adopted in wireless communication technologies [16,17,18,19]. IRS-based communication has also adapted different ML techniques for performance enhancement. The categories of ML applied in IRS are classified as deep learning (DL), reinforcement learning (RL), supervised learning (SL), unsupervised learning (UL), and federated learning (FL). Results in the previous studies demonstrated that ML-based methods have a comparable performance with the conventional methods while reducing the computational complexity [16,20]. Wireless communication technologies can be revolutionized by the power of ML. Recently, deep learning (DL)-based IRS technology can help to better extract the inherent relationship between the input–output signals and achieve a more reliable channel estimation, as shown for OFDM and mmWave massive MIMO [21,22] compared to the conventional or traditional model-based approaches. A DL-based channel estimation for an IRS-aided system was proposed in [23], where the authors proposed a single IRS system while considering MISO-OFDM. In [7], convolutional neural networks (CNN) for estimating the direct and cascaded channels sequentially were proposed. However, this design may suffer from the error propagation issue, since the estimated direct channel is used to construct the estimated cascaded channel.

In the literature, some survey papers covered IRS-based technologies [7,24,25,26]. Liu et al. [7] provided fundamental principals of IRS communication and interactions with electromagnetic signals. Next, the authors surveyed a performance analysis of the multi-antenna assisted IRS system, and beamforming and resource allocation techniques. Finally, a part for ML-based IRS communication and some direction integrating IRS with other technologies were provided. The key technologies that can be integrated with IRS are non-orthogonal multiple access, physical layer security, simultaneous wireless information and power transfer, unmanned aerial vehicles, and autonomous driving vehicles. The authors in [24] provided a survey of IRS technology that was focused on hardware design and implementation. In addition, basic concepts, the channel model, physical layer design implementations issues, AI-based solutions for IRS, and system deployment challenges were provided. The study in [25] conducted a survey on the IRS operation principle, metasurface fundamentals, IRS implementation structure, field-programmable gate-array-based IRS, and future research directions for IRS. Another study in [26] focused on a state-of-the-art survey of IRS-assisted technology below a 10 GHz frequency. The authors surveyed the IRS reflecting antenna array operation principle using microstrip patch antennas and adaptation in a wireless communication system. In addition, the authors discussed metasurface properties and reflections for metasurfaces. ML methods are considered to be a powerful tool in solving different problems in wireless communication, and will dominate the future research trend. Thus, this survey is entirely dedicated to reporting ML-based IRS works for the readers. The contributions of the paper can be summarized as follows:

- In the beginning, we give a complete insight into IRS technology. We provide a comprehensive introduction of the IRS technology, including its structure, working principle, and advantages.
- ML-based approaches are provided in a systematic way to understand the state-of-the-art research. We categorize different ML techniques as DL, RL, SL, UL, and FL. Each related paper is described in a comprehensive way by placing them into one of the categories. The problems that are related to IRS system using ML are described as channel state information (CSI), phase shift estimation, signal detection, beamforming, optimization, spectral efficiency, and privacy protection or security.
- Finally, we give some of the future research scopes that are further needed to be investigated by combined IRS and ML approaches.

The organization of the paper is as follows. Section 2 describes the IRS technology fundamentals. Section 3 describes the different ML methods used in the IRS communication system, some future research directions are described in Section 4, and Section 5 concludes the paper.

## 2. IRS Technology Fundamentals

In this section, we introduce typical IRS hardware architecture and the fundamental working principles.

### 2.1. IRS Hardware Architecture and Its Working Principle

Based on the meta-surface, the implementation of IRS hardware is digitally controllable, and is made of two-dimensional meta-material [27]. In order to operate in the subwavelength frequency of interest, the meta-surface is a planar array that consists of a large number of meta-atoms with electrical thickness [28]. The individual signal response, such as a reflection, amplitude, and phase shift of the IRS meta surface atom element, can be changed by properly designing the elements, including the geometrical shape (e.g., square or split-ring), size/dimension, orientation, arrangement, and so on. The reflection coefficient of each element should be tunable to cater to dynamic wireless channels arising from the user (UE) mobility in the wireless communication system. Three main approaches are introduced in the literature for controlling IRS reflection mechanical attenuation (e.g., mechanical translation and rotation), functional materials (e.g., liquid crystal and graphene), and electronic devices (e.g., positive-intrinsic-negative (PIN) diodes, field-effect transistors (FETs), micro-electromechanical system (MEMS) switches, etc.). Among them, electronic devices are widely adopted because of their fast response time, low reflection loss, and low energy cost. Finally, dynamically adjustable reflection coefficients are needed to create the IRS elements, and it is essential to link them to the network to acquire knowledge of the exterior communication environment in order to qualify its real-time adaptive reflection [29].

**Figure 1:**
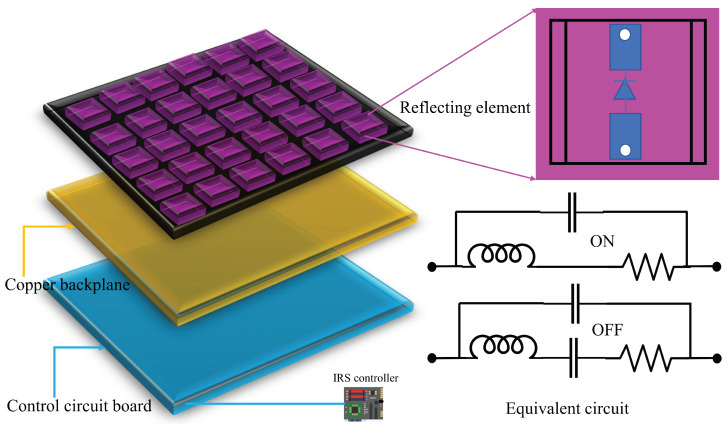
The architecture of reconfigurable reflecting element based on PIN diode with different layers.

One typical architecture of an IRS device is illustrated in Figure 1, where the IRS consists of a smart controller and three layers. The first layer is made up of a large number of reconfigurable metallic patches that are printed on the dielectric substrate to directly manipulate the incident signals. Behind this layer, a copper plate is usually used to avoid the signal energy leakage during IRS’s reflection. A control circuit board excites the reflecting element and is responsible for adjusting the reflection amplitude/phase shift of each element. In addition, the reflection adaption is triggered by a smart controller attached to the IRS. Moreover, the controller between BS and UE is accomplished by a field-programmable gate array (FPGA), which also works as an entrance to communicate and coordinate with other network parameters (e.g., base stations (BSs), access points (APs), and UE terminals) by different wireless links for low-rate information interchange between them.

Figure 1 shows the equivalent circuit as a sample of a separate element structure where each element is embedded with a PIN diode in the middle point of the element. The PIN diode can be changed to either the “ON” or “OFF” state by regulating various biasing voltages to the PIN diode through a direct-current feeding line, which allows the element to result in a phase-shift difference in π in the coming signal [30]. Setting up the corresponding biasing voltages by the smart controller, different phase shifts of IRS elements can be realized separately. According to [30], the changing frequency can be up to 5 megahertz (MHz) in the PIN diode element, which is equal to a changing time of 0.2 microseconds (μs). This is much smaller than the typical channel coherence time, which well matches with the application of a mobile with time-varying channels. Besides tuning the phase shift, extra control of the reflection amplitude of every element of IRS gives much greater flexibility in reshaping the reflected signal to obtain different communication objectives successfully. Additionally, this provides an effective way to trade off the relationship between the cost of hardware and the performance of the reflection. In practice, the amplitude control is basically more cost-effective than phase control and there are many ways to obtain amplitude adjustment for the IRS network. One of the common processes is to adjust the load resistance in every IRS element [31].

Consider this as an example: a particular location of the incident energy of the signal is decreased as heat by variation in the resistance of every element of IRS, and then an effective scale of the reflection amplitude in [0, 1] is achieved. This is almost the same as the work of a passive radio frequency identification tag that can regulate the reflected signal power strength via changing its load impedance for data modulation. For optimizing the reflection design, it is required to have independent control of the amplitude and phase shift of every element of IRS. However, this requires a more intelligent hardware implementation (e.g., the design of a multilayer surface [32]) than the aforementioned reason due to the individual control only. Ideally, the amplitude and phase shift per element can be individually and randomly tuned for IRS reflection.

### 2.2. IRS Reflection

**Figure 2:**
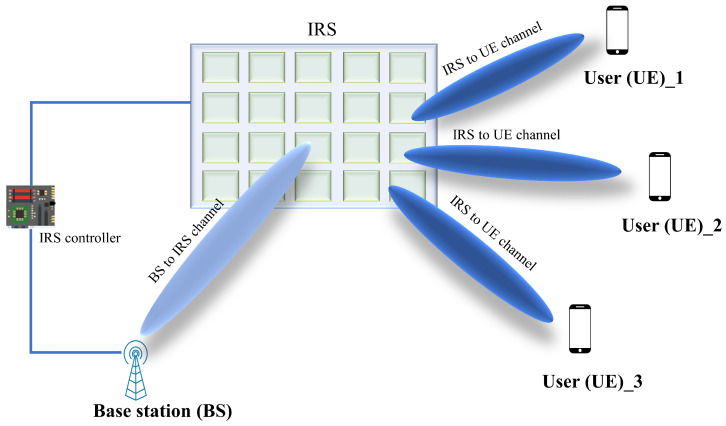
IRS-aided wireless communication system with BS and UE channel link.

Figure 2 shows the channel model design of IRS-based wireless communication where the channels from the BS to the UE through each element of the IRS are constructed with three components. They are the channel link between BS and IRS, the reflection of IRS elements, and the channel link between the IRS and UE. Such a composite channel behaves differently from the conventional point-to-point direct channel. However, from the BS, each element of the IRS receives the superposed multi-path signals and then scatters the associated signal with an adaptable amplitude and/or phase, such as a single point source, which leads to a “multiplicative” channel model.

We can express the complex reflection coefficient of the reflected signal by the *n*-th element of the IRS mathematically as follows:

(1) $$h_{n}=\left(\beta_{n}e^{j\theta_{n}}\right)x_{n},n=1,\ldots N,$$

where $h_{n}$ denotes the reflected signal of the nth element, $x_{n}$ is the incident signal to the IRS, *N* represents the total number of the reflecting elements at the IRS, and $\beta_{n}$ and $\theta_{n}$ present the Equations (1) and (2) with the reflection coefficient and control the reflected signal amplitude and phase, respectively [33]. By using the appropriate designs for the IRS phase shifts, amplitude, or both, a certain metric objective, such as the system achievable rate or the coverage, can be optimized by changing the channel environment. This is fundamentally different from conventional wireless communication research, where the design and optimization opportunities are confined to the pair of transceivers [24].

**Figure 3:**
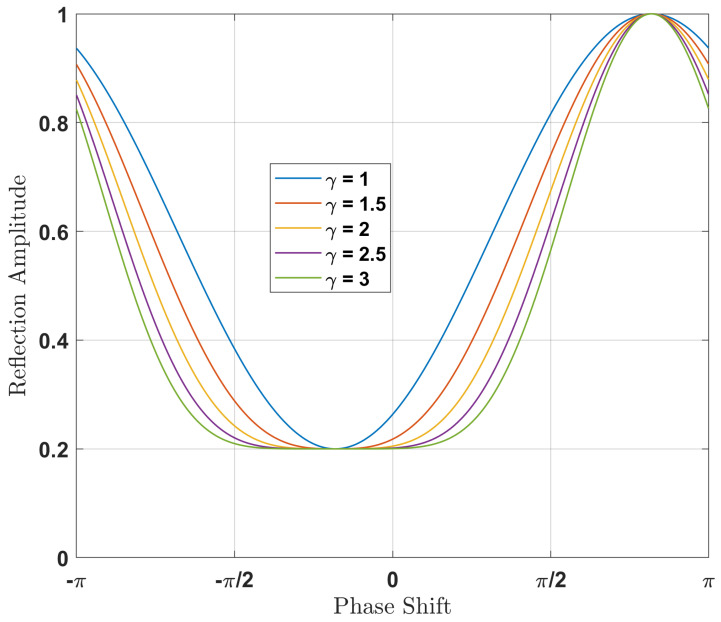
The phase shift model and its different parameters.

Moreover, according to the design of the IRS-aided wireless systems, for the fundamental relationship between the reflection amplitude and phase shift, we can assume an analytical model for the phase shift. The model is generally applicable to a variety of semiconductor devices used for the application of the IRS. We consider the phase shift and the corresponding amplitude $L_{n}=\beta_{n}\left(\theta_{n}\right)e^{j\theta_{n}}$ with $\theta_{n}\in \left[-\pi ,\pi \right]$ and $\beta_{n}\left(\theta_{n}\right)\in \left[0,1\right]$, respectively. Therefore the reflection amplitude $\beta_{n}\left(\theta_{n}\right)$ can be expressed as follows:

(2) $$\beta_{n}\left(\theta_{n}\right)=\left(1-\beta_{min}\right){\left(\frac{sin(\theta_{n}-\phi )+1}{2}\right)}^{\gamma }+\beta_{min},$$

where $\beta_{min}\geq 0$, ϕ≥0, and γ≥0 are the constants related to the specific circuit implementation. Figure 3 shows that the $\beta_{min}$ is the minimum amplitude, ϕ is the horizontal distance between −π/2 and $\beta_{min}$, and γ controls the steepness of the function curve. Note that, for γ=0, (2) is equivalent to the ideal phase-shift model, i.e., $\beta_{n}\left(\theta_{n}\right)=1,\forall n$. In practice, IRS circuits are fixed once they are fabricated and these parameters can be easily found by a standard curve fitting tool [34].

### 2.3. Channel Model of IRS

**Figure 4:**
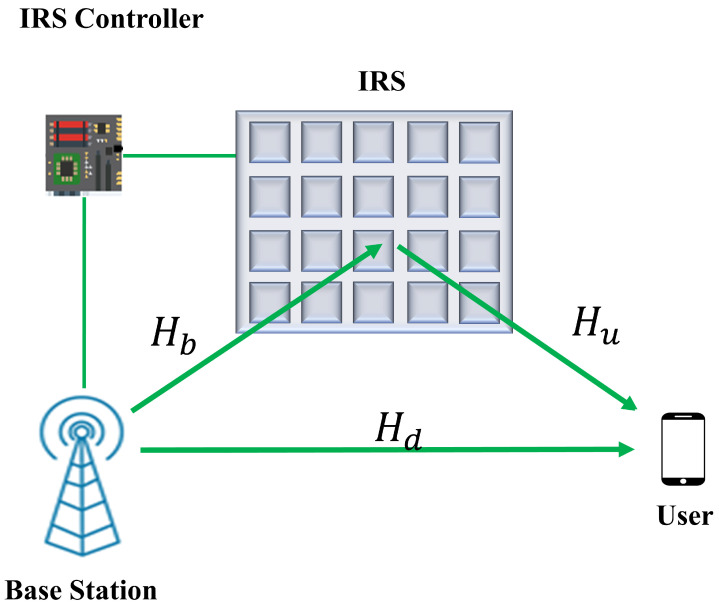
IRS-based communication model for base station to user link scenario.

We consider the system model shown in Figure 4, where a BS communicates with a UE using an IRS-assisted network. It is assumed that the base station has *M* uniform linear array antennas and the IRS has *N* reflective elements. One user is considered in the services area having a single antenna. The received signal via IRS can be defined as [10]:

(3) $$y_{irs}=h_{u}\Psi H_{b}x+n$$

where $y_{irs}$ is the received signal at the UE, $x\in C^{M\times 1}$ is the transmitted signal, $H_{b}\in C^{N\times M}$ is the channel matrix from BS to IRS, $h_{u}\in C^{1\times N}$ is the channel vector from IRS to UE, and $n∼\mathrm{CN}(0,\sigma^{2})$ is the additive white Gaussian noise (AWGN) at the user. Ψ= diag(z) $\in C^{N\times N}$ is a diagonal matrix representing the phase shift values with $z={\left[\beta_{1}e^{j\theta_{1},}\beta_{2}e^{j\theta_{2}},\ldots \beta_{N}e^{j\theta_{N}}\right]}^{T}\in C^{N\times 1}$. The total channel with direct communication can be formulated as [35]:

(4) $$y_{t}=h_{u}\Psi H_{b}x+H_{d}x+n$$

where $H_{d}\in C^{1\times M}$ is the direct channel between BS and UE. The BS-IRS channel $H_{b}$ can be written as follows:

(5) $$H_{b}=\sqrt{\frac{MN}{P_{1}}}\sum_{p_{1}=1}^{P1}\alpha_{p}^{1}t\left(\beta_{p}^{1},\theta_{p}^{1}\right)r\left(\beta_{p}^{1}\right)$$

where $\sqrt{\frac{MN}{P_{1}}}$ is the normalization factor, $P_{1}$ is the number of paths between BS and IRS, $\alpha_{p}^{1}$ is the complex gain for path *p*, $t\left(\beta_{p}^{1},\theta_{p}^{1}\right)\in C^{N}$ are the array response vectors related to IRS, and $r\left(\beta_{p}^{1}\right)\in C^{M}$ are the array response vectors of BS. The array response vectors can be expressed as follows:

(6) $$t\left(\beta ,\theta \right)=\frac{1}{\sqrt{N}}[1,\ldots ,e^{j\frac{2\pi }{\lambda }d_{r}\left(n_{1}\cos\left(\theta \right)\sin\left(\beta \right)+n_{2}\sin\left(\theta \right)\right)},\ldots ,e^{j\frac{2\pi }{\lambda }d_{r}\left(\left(N_{1}-1\right)\cos\left(\theta \right)\sin\left(\beta \right)+\left(N_{2}-1\right)\sin\left(\theta \right)\right)}{]}^{T},$$

(7) $$r\left(\beta \right)=\frac{1}{\sqrt{M}}[1,\ldots ,e^{j\frac{2\pi }{\lambda }d_{a}\left(m\sin\left(\beta \right)\right)},\ldots ,e^{j\frac{2\pi }{\lambda }d_{a}\left(\left(M-1\right)\sin\left(\phi \right)\right)}{]}^{T},$$

where $d_{r}$ is the elements spacing for IRS, $d_{a}$ is the antennas spacing for BS, λ is the signal wavelength, and $0\leq n_{1}<N_{1}$ and $0\leq n_{2}<N_{2}$ denote the horizontal and vertical indices of IRS elements. For indexing the BS antenna, *m* is used, where 0≤m<M. The channel matrix between the IRS and UE can be expressed as follows:

(8) $$h_{u}=\sqrt{\frac{N}{P_{2}}}\sum_{p_{2}=1}^{P2}\alpha_{p}^{2}r_{2}\left(\beta_{p}^{2},\theta_{p}^{2}\right),$$

where $\sqrt{\frac{N}{P_{2}}}$ is a normalization factor, $P_{2}$ is the number of paths between IRS and UE, $\alpha_{p}^{2}$ is the complex gain for the *p*-th path, and $\beta_{p}^{2}$ and $\theta_{p}^{2}$ are the azimuth and elevation of angle of departure, respectively.

The cascade channel from BS to UE can be expressed as follows:

(9) $$H_{ca}=\sqrt{\frac{MN}{P_{1},P_{2}}}\sum_{p_{1}=1}^{P1}\sum_{p_{2}=1}^{P2}\alpha_{p}^{1}\alpha_{p}^{2}\times diag\left(r_{2}\left(\beta_{p}^{2},\theta_{p}^{2}\right)\right)t\left(\beta_{p}^{1},\theta_{p}^{1}\right)r\left(\beta_{p}^{1}\right)$$

### 2.4. IRS Based Communication Advantages

**Figure 5:**
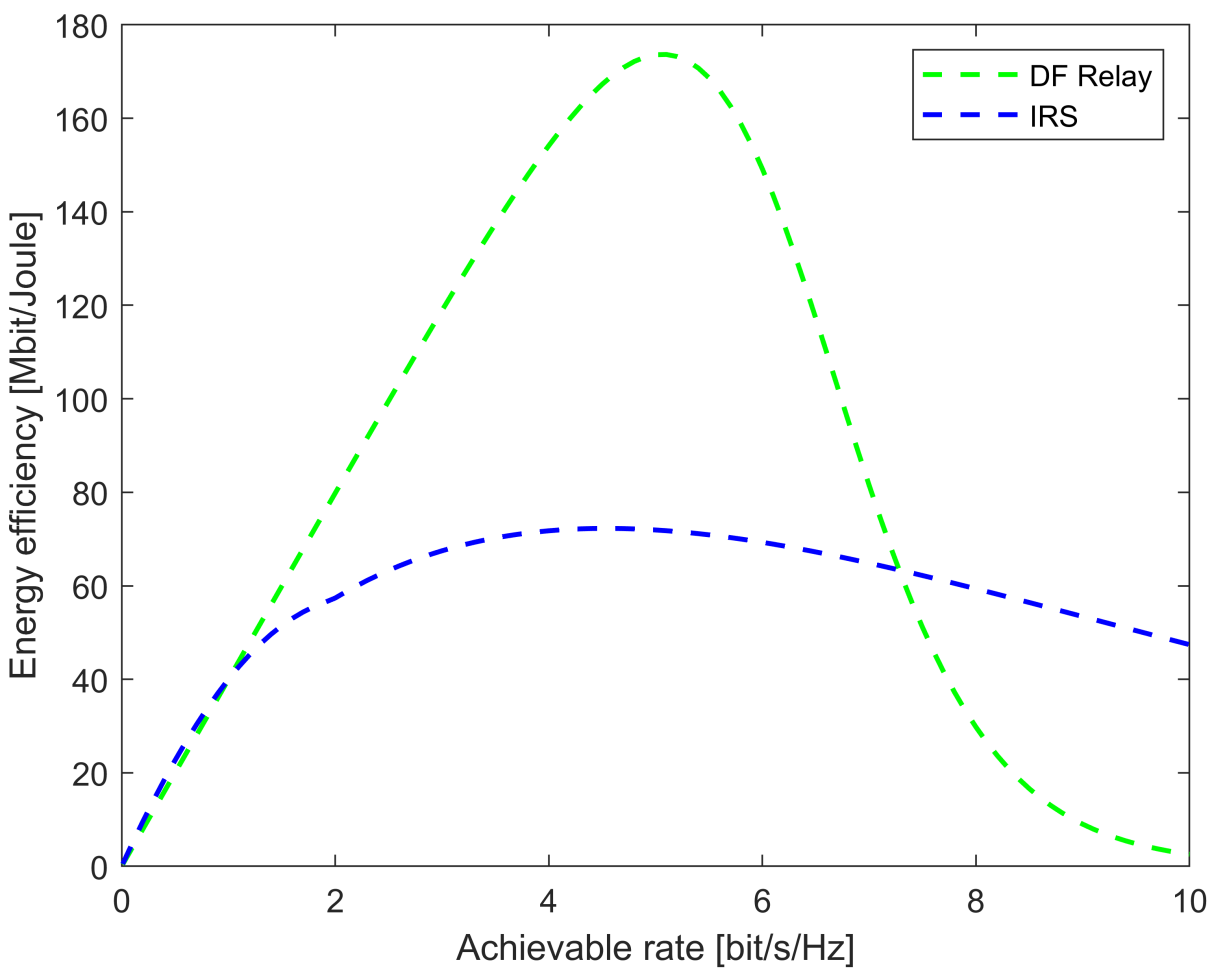
The comparison between energy efficiency and data rate for DF relay and IRS.

Finding a clear line-of-sight communication channel is a big challenge for wireless communication. In a practical scenario, many obstacles can make the transmitted signal very weak, and then interruption of the service can occur. Figure 5 shows the comparison between the energy efficiency and data rate for the IRS and decode-and-forward (DF) relay system [36]. It is evident that IRS can provide a higher data rate in low-energy consumption compared to the DF relay. Some of the striking advantages can be listed as:

- IRS can provide an alternative path where direct communication is not possible. A virtual path is established dynamically when the communication takes place between the transmitter and receiver [29].
- IRS devices are passive in nature, which implies less power consumption compared to relay communication. The amplification and forwarding of an incoming signal are possible without employing power amplifiers [37]. Instead, the signal phase shift is controlled by the reflecting elements to direct the signal to the UE.
- Millimeter wavelength communication can address the bandwidth shortage problem for 6G communication. However, the path loss is higher than other low-frequency bands [38]. IRS can improve the communication to gain a better performance.
- IRS can be utilized to compensate for the channel rank condition in an environment suffering from a rank deficiency issue [39].
- The propagation of EM waves can be reconfigured in a software-controlled fashion, which can turn the probabilistic wireless channel model into a deterministic model [40].
- As the IRS is based on the reflection of signals with directed beamforming, Ralyleigh fast fading is converted to Rician slow fading.
- IRS can provide an effective solution for both the co-channel and inter-channel interference of wireless communication [29].

## 3. Machine Learning for IRS-Assisted Communication Systems

The ML technique is a powerful technology and has achieved remarkable interests in wireless communications due to its learning capability and large search-space [41,42,43]. In this section, we review the existing research contributions related to ML-based IRS wireless communication with its application and challenges. In addition, ML-based IRS systems and potential opportunities for future research community are presented. Table 1 shows a comparison between some recent ML-based technologies for IRS-assisted wireless communication.

**Figure 6:**
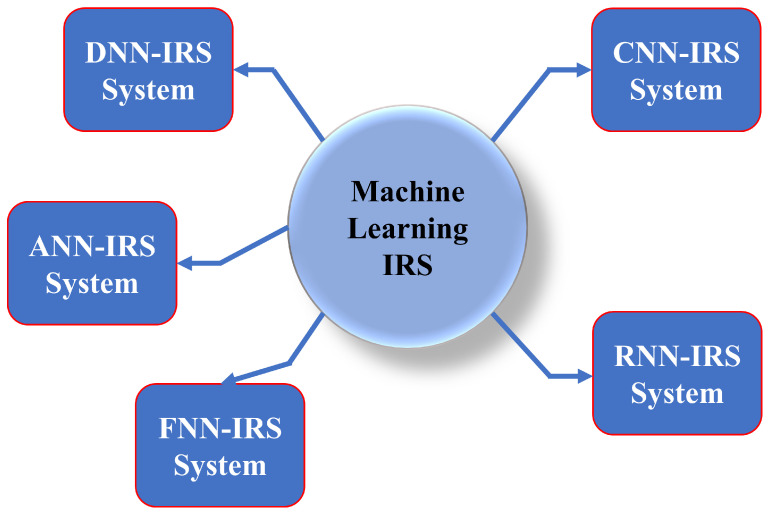
Taxonomy of ML-based IRS system.

ML is related to the portion of science that studies the theory and characteristics of learning algorithms, their performance, and associated systems. ML is a wide multidisciplinary area that draws concepts from a variety of domains, including information theory, AI, statistics, optimal control, and optimization theory. In addition, it makes new ideas for other scientific, mathematical, and engineering fields [44,45,46,47]. Due to the deployment in different field applications, ML has touched nearly every scientific subject and has had a significant effect on research and society [48]. Some recent application fields of ML are autonomous systems, suggestion engines, informatics, data mining, and recognition systems [49]. The ML technology consists of two phases: training and decision making. In the training phase, a dataset is used to train and understand the model of the system. During the decision-making process, the trained model is employed to derive the projected output for every new input given to the system. The taxonomy of ML involves various subfields, such as RL, UL, and SL [50]. A detailed classification of an ML-based IRS system is described in the next section by the overview of Figure 6. We also summarize machine-learning approaches for IRS-based communication in Table 2.

### 3.1. Deep Learning for IRS-Enhanced Communication Systems

For the revolution of communication systems, DL is a potential and important technique. As a result of its potential learning capabilities, DL can be applied in various areas of IRS-enhanced wireless networks [16,51,52]. In MIMO communication, the timely acquisition and appropriate CSI play a vital role in the wireless communication system. Due to the large number of antennas in a massive MIMO communication system, the estimation of CSI becomes more difficult and complex [53]. To overcome this drawback, DL-based research has been conducted by many researchers for estimating CSI, especially for DL-based IRS communication systems [54]. The authors in [35] proposed a DL method for training the IRS reflection matrices from sampled channel knowledge without any command of the IRS geometrical array for estimating the large number of unspecified parameters that are created by IRS. The key idea is to extract environment descriptors that capture information about the multi-path signature of a signal travelling towards IRS. This information helps to train the DL model to estimate the channel parameters. In [55], authors proposed a DL algorithm for the enhancement of the key generation rate (KGR) in the physical layer key generation by using a single antenna UE time division duplexing mode system. The authors proposed KGNet for frequency band feature mapping to construct a reciprocal channel feature between communication parties. To estimate the compressive CSI for IRS-assisted mmWave systems with a low training overhead, the authors in [23] introduced a deep denoising neural network. The proposed DL method can jointly process real and imaginary parts of the channel matrix. The simulation results provide a robust result for SNR = 10 dB, with different numbers of multipath signal components. In this way, for estimating the CSI of an IRS-assited wireless network, the DL method can be adopted. In addition to the above applications of DL in IRS-enhanced wireless networks, to estimate the mapping between a UE position and the IRS phase configuration to maximize the received SNR, a deep neural network (DNN)-based approach in the indoor communication environment was proposed in [56]. The proposed DL architecture consists of five input layers, three hidden layers, and an output layer. The hidden layers are mainly responsible for the input–output mapping the user position and optimal IRS phase configuration. Moreover, for the optimal IRS phase shift configuration, DL can also be applied. The authors in [57] proposed a DL-based algorithm for optimally designing the phase shift of the IRS by training in an offline stage. As the number of reflecting elements increases, the effective gain of the reflecting path increases. In addition, an increase in the number of antennas in the user can enhance the performance. In addition, the DL-based IRS wireless networks can be deployed for signal detection. The signal estimation and detection in the IRS-assisted wireless networks were carried out in [58]. For estimating channels and phase angles of a reflected signal received by an IRS, a DL-based approach was introduced. The bit-error-rate is also improved by the application of the DL method in an IRS-based wireless communication system.

### 3.2. Reinforcement Learning for IRS-Enhanced Communication Systems

**Figure 7:**
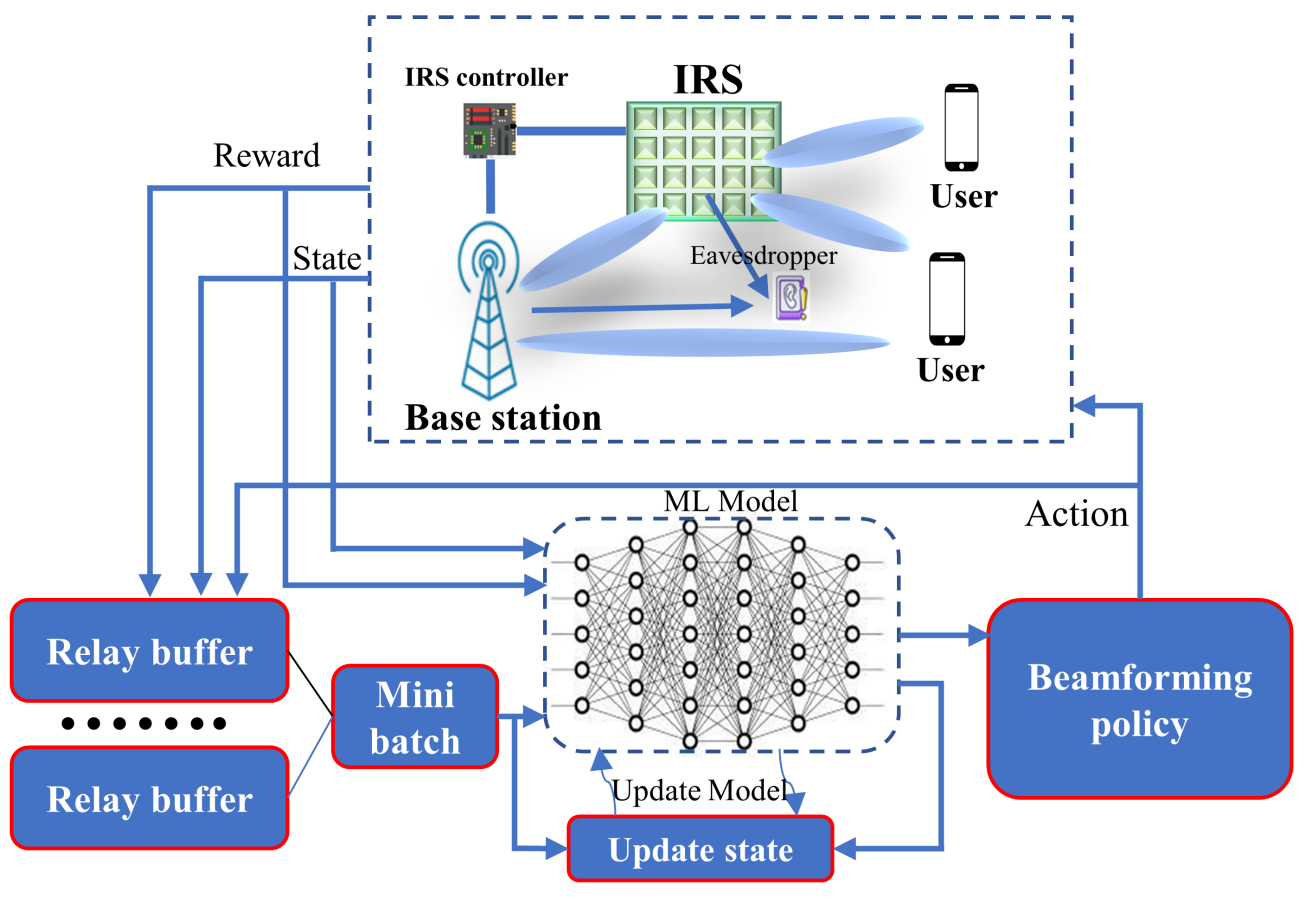
RL-based IRS-enhanced communication system.

RL is constructed to transform the AI field. In addition, it represents a next step with regard to the creation of autonomous systems with a higher-level knowledge of the visual world. Currently, RL is also applied to the field of robotics, allowing control policies for robots to be learned directly from camera inputs in the real world [59]. The learning capability of the RL model exploits learning from the environment, learning from the feedback of UE, and learning from its mistakes, which may mitigate the challenges encountered in conventional IRS-enhanced wireless networks, and thus the performance can be improved. In recent years, several works related to RL-based IRS wireless communication have been carried out to enhance the communication performance, focusing on 6G network application [60,61,62]. Figure 7 shows a typical application scenario for RL-based communication. Each user sends the feedback through a relay buffer, which is used to update the beamforming policy by the base station. In [63], the authors proposed deep deterministic policy gradient (DDPG)-based models in the IRS wireless network with the multiple input single output (MISO) communication system. Based on the simulation result, the IRS-NOMA downlink outperforms IRS orthogonal multiple access (OMA) downlink transmission significantly. However, the complexity of the proposed methods increases exponentially as the number of reflecting element increases.

The phase shifts of the IRS structure are mainly of discrete form, so the deep Q-network (DQN) learning model is appropriate to mitigate the difficulty of the phase shift design. For acquiring the whole advantages of an IRS wireless communication system with the aid of an RL algorithm, the architectures of the joint transmit and passive beamforming of an IRS-assisted communication system were considered in MISO systems [64,65], OFDM-based systems [66], wireless security systems [67], and millimeter wave systems [68]. Moreover, the RL-based algorithm is susceptible to simultaneously design the alternating optimization (AO) method for the transmit beamforming at the BS and the passive beamforming at the IRS device. For more details in [64], the authors proposed a DDPG-based algorithm by utilizing the sum rate as instant rewards for training the model to gain a maximum throughput. In the applied model, the successive transmit beamforming and IRS phase shift were cooperatively optimized with less complexity. A deep RL approach in [65] was proposed for IRS phase shift design. Under the Rician fading channel, the proposed approach almost achieves an upper bound in terms of the received SNR value. By using the direct optimizing interaction matrices from the sampled channel knowledge in [66], the authors applied a deep RL-based algorithm for maximizing the achievable communication rate in the IRS wireless network. The only single beam was used for training episodes in the applied deep RL algorithm. Thus, the proposed algorithm can avoid the data collection phase during the training period, which reduces the computational time. RL-based approaches can ensure security in IRS-based communication, which was reported in [67]. The optimal beamforming for both the base station and IRS reflection maximizes the worst-case secrecy rate. The proposed scheme can enable a secrecy rate with a satisfactory quality of service. In [68], authors proposed a deep RL-based algorithm that acquired a maximum throughput by estimating the perfect and imperfect CSI and modeling a return distribution for each state-action pair, and a univariate regression algorithm was also proposed, which modeled the inherent randomness interconnection between the IRS and communicating environment. In addition, a post-decision state and prioritized experience replay schemes were used to enhance the learning efficiency and secrecy performance.

### 3.3. Supervised Learning for IRS-Enhanced Communication Systems

**Figure 8:**
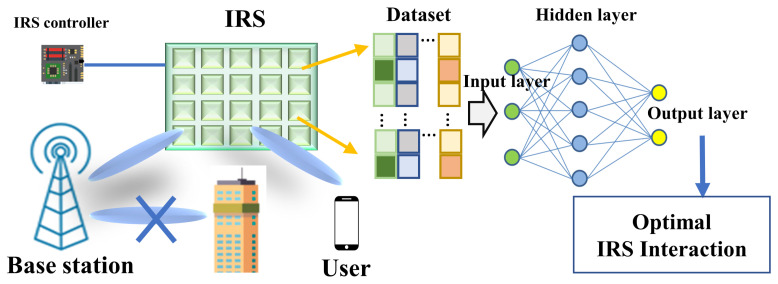
SL-based IRS-enhanced communication system.

In the presence of a supervisor, the type of learning that recognizes the system parameters is known as SL. In this learning, the collection of data is needed for an algorithm that is combined with output and input information. Depending on the input and output data connection, the model can be built up. After that, a fresh data set is input into the model to obtain a prediction result [69,70,71]. SL is one of the strongest branches of ML, which includes regression, decision tree and random forest, K-nearest neighbors (KNN), support vector machines (SVM), and Bayes classification. This powerful model can be applied to various challenging fields, such as spectrum sensing [72], prediction of traffic/QoE [73], channel/antenna sorting [74], and association of network [75]. In recent years, owing to the benefits of a low complexity and faster speed, SL algorithms can be applied to solve the associated difficulty with enough training data in the IRS-empowered wireless communication system. Figure 8 depicts the SL-based IRS communication system. The signal generated from the base station is first transmitted to IRS elements. Next, the reflected signal from IRS is captured at the user to create a dataset. The dataset is used for training to create an SL-based model for an optimal IRS interaction, as shown in Figure 8. In [76], authors proposed a SL convolutional neural network (CNN) model in the MISO system for obtaining the sum-rate maximization in the IRS wireless communication system. The CNN model is deployed in the IRS, where the input is an incident RF signal and the output is the set of interfering users. More than a 99% accuracy was achieved by the proposed model. The OFDM-based single receiving antenna system model has been utilized in the IRS network to achieve a maximum performance rate with the SL algorithm [35,77,78]. The study in [77] proposed an ordinary differential equation (ODE)-based CNN model for IRS-based communication. ODE-based methods can be used to describe the latent relation between different layers in a neural network. The performance analysis shows that ODE-based CNN is always superior compared to a standard CCN network. The authors in [78] proposed an algorithm that leverages previous channel information to improve the quality of an optimal IRS interaction. The authors of [79] proposed a SL algorithm in the OFDM-based single input single output (SISO) system to obtain a maximum performance in the IRS network. The MIMO system can obtain the maximum output performance with the assistance of the SL-CNN model, where the received pilot signals were used as the input data for the ML model [23,80]. The study conducted in [80] proposed a twin CNN architecture for both direct and cascade channel estimation using pilot signals. The proposed method can tolerate a four-degree displacement in the user location. In addition, a SL-based IRS wireless network communication system was carried out in the [78,81,82,83], where most of the papers mentioned the increase in the achievable rate maximization.

**Table 1:** Comparison between ML-based technologies for IRS-assisted wireless communication.

References	ML Model Architecture	Major Contributions	Remarks
[84]	DNN with three full layers	Phase reconfiguration	Performance is close to the perfect CSI-based approach. The pilot signal overhead is reduced
[85]	Multi-layer perceptron (MLP) with eight hidden layers, ReLU activation function	CE by normalized mean squared error algorithm	Performance improves with higher signal-to-noise ratio (SNR)
[23]	Complex-valued DnCNN	Compressive sensing-based broadband CE algorithm	Robustness makes it possible for application in different SNRs without repetitive training
[86]	Deep-learning-based phase shift control (D-PSC), fully connected layers	Find out optimal phase shifts maximizing data rate	Data rate more than 25% over the conventional phase shift control schemes using the same pilot resources
[54]	CNN with three convolution layers	Predict the optimal IRS phase shift	Can converge to near-optimal data rates using less than 2% of the total number of receiver locations
[87]	Deep-RL	Decaying-DQN-based algorithm	Proposed system significantly reduces energy dissipation by integrating IRSs in UAV-enabled wireless networks
[88]	ML-inspired algorithmic framework	Cross-entropy optimization	Proposed method can simultaneously optimize transmit and reflecting beamforming in an IRS-assisted wireless system
[89]	ML framework	Optimization-driven DDPG algorithm	Proposed model can improve both convergence and reward performance compared to conventional model-free learning scheme
[90]	Fully-connected DNN model	Spectral efficiency problem	Proposed model has less computational complexity and does not require any computational load for data labeling
[91]	Neural network model	IRS-aided localization calculation	Proposed system requires multiple APs and a large number of fingerprint grid samples and then acquires great localization results
[83]	DNN with three hidden layers	Beam management (BM) classification for mmWave networks	Gained highly efficient BM with remarkably attenuate system overhead
[52]	Artificial neural network (ANN) with 10 layers, ReLU activation functions	ANN data-driven approaches for optimization	Proposed model can be trained to learn virtually any input–output map [92]
[93]	CNN with three conventional layers, ReLU activation functions	CE using deep denoising algorithm	Proposed method can use optimal minimum mean square error estimator with channel probability density function
[94]	Recurrent neural network (RNN) model, ReLU activation functions	CE using single and multi-scale RNN algorithm	Model enhanced flexibility of overall network to obtain better generalization and fitting capabilities

**Table 2:** Machine learning approaches for IRS-based communication.

IRS Communication Problem	ML Approach	Developed Model
Channel estimation	DL, SL, RL, FL	deep multi-layer perceptron, ChannelNet, CV-DnCNN, DReL, CDRN, ODE-CNN, KGNet
Signal detection	DL	DeepIRS, CNN, SVM
Phase shift configuration and beamforming	DL, RL, SL, FL, UL	DQN, DNN, DL-RNN, DQN, DeepMIMO, LPSNet
Security	DL, FL	DRL, CNN
Resource allocation	DL, FL	DNN, AirFL

In [81], a hybrid precoding architecture was proposed for THz communication for IRS-based communication. An SL-based approach was adapted to optimize the hybrid precoding problem. It has been shown that the proposed method has a similar performance to the traditional hybrid precoding algorithm. The authors in [82] proposed an RIS-assisted drone communication network where the drone is connected to the base station via IRS. An SL-based approach was implemented to find the consistent communication link between the base station and drone. The study in [83] proposed an ML-based beam management framework for IRS-assisted mmWave networks. Environmental and mobility awareness were used to maximize the beamforming accuracy.

### 3.4. Unsupervised Learning for IRS-Enhanced Communication Systems

The algorithm helps to find out the error level for every inspection by correctly recognizing the input and output data without any assistance by a supervisor called an UL machine [95]. In brief, the UL technique constructively finds the data connections to form a cluster and obtain an unlabeled input dataset [69]. UL methods do not depend on prior knowledge and this learning technique is not data-hungry. Several UL algorithms have been developed in the recent research community [96], such as clustering of K-means, maximization of expectation, principal component analysis (PCA), and independent component analysis (ICA). These proposed UL algorithms can be deployed in the IRS-enhanced wireless communication to improve the difficulty issue, such as the deployment of BS, UE clustering/association of UE [97], detection of network state [98], aggregation of dataset [99], and cancellation of interference [100]. In [101], authors proposed IRS-assisted UL-CNN, a feedforward neural networks (FNN) algorithm in the MISO communication system with multiple UEs to acquire the maximum sum-rate. To obtain the maximum spectral efficiency, a UL-based learning phase-shift neural network (LPSNet) algorithm was proposed in [90]. The maximization problem of spectral efficiency was formulated and a small number of hidden layers were used in the ML architecture to solve the problem. The proposed algorithm was tested with 16×2 MIMO configuration.

### 3.5. Federated Learning for IRS-Enhanced Communication Systems

**Figure 9:**
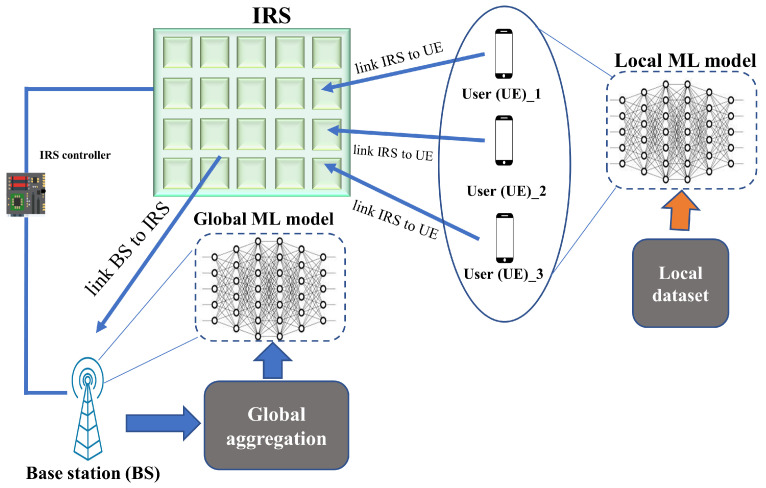
FL-based IRS-enhanced communication system.

In ML, FL has become the main point in the large-scale area and distributed optimization due to the exploration of training statistical models by a direct route on distant devices [102]. In the FL, the inaccessibility of personal data is no longer a problem. The FL algorithm is learned at the edge in allocated networks. The FL model can be deployed to design multiple IRS networks because of its privacy-preserving nature. In this network, IRS operates as a distributed trainer, trains its generated data, and also transfers it to local model parameters instead of the raw training dataset to an aggregating unit. In this way, FL can be learned in a decentralized manner for the deployment and design policy. Figure 9 shows an example of an FL-based IRS networking system. Each user has a local ML model trained by a local dataset that sends the learned parameter to the base station. Again, the base station trains a global model by having the parameters from users. In [103], authors proposed an IRS-assisted optimal beam reflection (OBR)-FL algorithm in the single receiving antenna communication system with multiple UEs to increase the data rate. The experimental results suggest that the achievable rate is similar to other centralized machine learning models and that there is no significant difference if the receiver number is changed. In [104], the authors proposed an air federated learning (AirFL) framework to solve resource allocation and device selection problems for aggregation accuracy enhancement and coverage rate improvement. According to the simulation results, the proposed model can converge faster, with a small training loss. In order to contribute to unmanned aerial vehicle (UAV) trajectory application, an MIMO system was designed with the proposed FL-CNN model, where the received pilot signals were used as the input data for the ML model [105]. For reliable channel estimation, the authors proposed that the model should be trained on 15 dB SNR and 5 bit quantization.

## 4. Future Research Trends for ML-Based IRS-Assisted Wireless Communication

**Figure 10:**
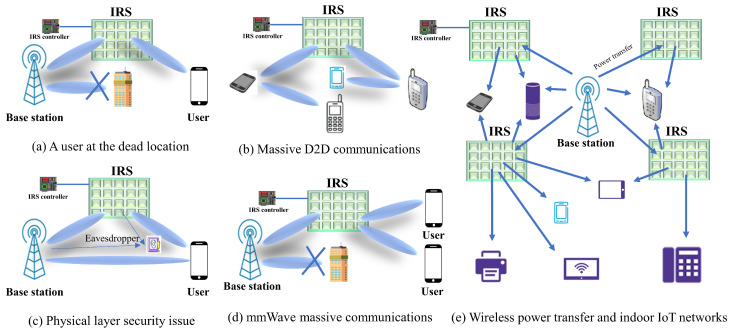
IRS-aided wireless communication applications.

ML-based models are efficiently deployed for wireless communication to enhance the service quality. The different application scenarios of an IRS-based wireless communication system are illustrated in Figure 10. Figure 10a shows a situation where a user is out of the line of sight due to an obstacle; in this case, IRS can help with communication. As shown in Figure 10b, massive devices can be implemented using IRS. Figure 10c shows that physical layer security can be enhanced using IRS with controlled beamforming. It is expected that mmWave massive communication can be implemented successfully using IRS as shown in Figure 10d. Figure 10e shows that IRS can be useful in wireless power transfer for indoor IoT networks. In this section, we describe some of future research scopes that combine ML and IRS.

### 4.1. Optimal Placement of IRS

The position of the reflecting surface is fixed and cannot be changed after implementation. Thus, it is necessary to have sufficient information for the performance of IRS in the environment before practical deployment. To obtain a maximum performance, the placement of the IRS device should be based on the operating performance. This is applied for both the indoor and outdoor placement of IRS. ML models can be employed to estimate the particular cases with optimized results [106]. As each of the installation cases may be different, new models can be effective to estimate the performance.

### 4.2. Dynamic Hybrid Beamforming

IRS devices can create controllable beamforming by reflecting the BS signal. The beamforming depends on the passive elements on the IRS device and can generate UE-specific formation. ML algorithms can be efficiently applied to estimate effective beamforming for the UE service. In addition, UE feedback can be added to make the dynamic beamforming for mobile UEs. New training data and models need to be investigated for optimal performance.

### 4.3. Data Collection and Model Training

Data collection is a crucial part of training ML models and the accuracy of the model depends on the data [107]. As biased data can result in imperfect models, data collection is a challenge for the deployment of ML-based techniques in IRS-based communication. The main estimation factors are signal detection, CE, and the beamforming design for the receiver. For the successful implementation of ML-based techniques, data collection algorithms can be a future research topic.

### 4.4. Constrained System Modeling

IRS-based communication can be employed in a constrained system or device to increase the performance without additional cost. Energy and time-constrained communication systems in industrial environments can be investigated by using ML methods. In addition, a good signal-to-noise ratio can be achieved for non-line of sight communication. A new investigation can be made to identify the advantages of IRS-assisted communication for such systems.

### 4.5. Channel State Characterization

Different models have been proposed to categorize the IRS-assisted communication, such as an independent diffusive scatter-based model, physics model, impedance network-based model, and tile and code-book-based framework [108]. Machine-learning-based CE is now undergoing research, and different techniques have been used to estimate channel modeling [109]. However, many proposed optimization techniques are computationally complex. New optimization techniques can be a scope for future research.

### 4.6. IRS for IoT Network

IoT devices are increasing exponentially due to their diverse applications and high demands [110]. The IoT and 6G technology are accelerating the industrial revolution 4.0. IRS-based wireless communication can increase the connectivity of IoT devices in both indoor and outdoor environments. As the IoT devices are placed in a scattered way, the wireless system performance is unpredictable, and, thus, ML-based algorithms can be adapted to mimic the environment behavior. Most IoT devices are sensor nodes that generate different data related to some physical values. Further research can shed light on the impact of IRS for IoT connectivity using ML methods.

### 4.7. Protection Against Eavesdropping

Security is a great concern in communication systems in recent years and physical layer security (PLS) has attracted considerable attention. PLS has an advantage over the cryptography technique. PLS has less computational complexity and the structure of 6G network is decentralized; thus, key management will be difficult for each device in the network [111]. As IRS creates beamforming, security during data transmission can be achieved. Different environment threats combined with ML techniques can be modeled, and appropriate actions can be taken. This approach can be investigated in future studies.

### 4.8. MmWave Communication

The mmWave is considered a promising technology for increasing the bandwidth demand in 6G communication [112]. Some studies have been performed using IRS-assisted mmWave communication, but more studies are needed to investigate the impact of IRS. Some research directions can be modulation techniques, antenna array architecture, scheduling algorithms, signal waveform, and the deployed IRS with ML methods.

### 4.9. EDGE Intelligence

Edge computing refers to the movement of computations on the network edge rather than in the server [113], and eventually transfers the processing on source proximity. Edge computing provides a low latency, less energy consumption, low cost, privacy, and bandwidth efficiency in the communication of UE to the server. ML-based edge computing can provide an optimized system for network function [114]. It is expected that IRS and ML can provide a better performance for edge computing, which needs to be investigated in the future.

### 4.10. Hybrid Communication Implementation

Visible light communication (VLC) can also be a supporting technology to enhance the RF bandwidth [115]. A VLC/radio frequency (RF) hybrid technology can open new areas of research using IRS-based communication. As an example scenario, underwater communication can be a candidate for a hybrid communication system. Optical communication [116] can be used for data transmission in deep underwater and RF communication can be integrated to transmit data to the server near the surface. As IRS is based on the reflection of signals, it will work on both optical and RF waves.

## 5. Conclusions

In this paper, we have provided a comprehensive survey on ML-based IRS enhanced wireless communication technology for solving different problems. We have surveyed different ML-based IRS communication system model architectures, such as SL, UL, FL, and RL, with extensive discussion. Finally, we have provided some future research opportunities, challenges, and applications of ML-based IRS communication. We hope that the ML-based IRS system will contribute to the efficient direction and design of a high-performance system for the next generation wireless communication.

## Data Availability

Not applicable.
